# Sex hormone dysregulation after traumatic brain injury: interactions with sleep disturbances and seizure susceptibility

**DOI:** 10.3389/fnins.2026.1672744

**Published:** 2026-01-16

**Authors:** Isabella S. Elkinbard, Dana Ritterbusch, Oleksii Shandra, Rachel K. Rowe

**Affiliations:** 1 Department of Integrative Physiology, University of Colorado Boulder, Boulder, CO, United States; 2 Department of Biomedical Engineering, Florida International University, Miami, FL, United States

**Keywords:** concussion, hormone replacement, post-traumatic seizure, sex differences, sleep-wake disturbances

## Abstract

Each year, approximately 2.9 million people in the United States sustain a traumatic brain injury (TBI), many of whom go on to experience chronic secondary complications such as post-traumatic epilepsy (PTE) and sleep–wake disturbances. These outcomes arise from complex secondary injury processes, including neuroinflammation, oxidative stress, and disruptions in neuroendocrine signaling. While inflammatory and excitotoxic mechanisms have been extensively studied, growing evidence highlights sex hormone dysregulation—particularly involving estrogen, progesterone, and testosterone—as an important yet underrecognized contributor to post-TBI physiology. Clinical and preclinical studies indicate that TBI can alter systemic and brain-derived hormone levels, influencing neuroinflammation, glial activation, neuronal survival, and synaptic plasticity. These hormone-related changes have been associated with altered seizure susceptibility and disrupted sleep architecture, suggesting that sex hormone dysregulation may represent one interacting pathway influencing both outcomes. Additionally, the bidirectional relationship between epilepsy and sleep—where seizures disrupt sleep architecture and sleep loss increases cortical excitability—may further compound vulnerability after TBI. Given the heterogeneity of injury mechanisms and hormonal responses across individuals, these relationships remain incompletely understood but biologically plausible. This narrative review examines how TBI-related alterations in estrogen, progesterone, and testosterone may intersect with sleep regulation and seizure susceptibility. We summarize their physiological roles in the brain, evaluate how post-injury disruptions may shape chronic outcomes, and highlight how early identification of hormonal abnormalities could inform future research on therapeutic strategies. By addressing this understudied interface between endocrine, neural, and behavioral dysfunction, we aim to advance understanding of modifiable pathways that may contribute to long-term morbidity after TBI.

## Introduction

A traumatic brain injury (TBI) results from a forceful bump, blow, or jolt to the head, neck, or body, or penetration of the brain tissue from an external object. The initial damage, known as the primary injury, occurs immediately due to mechanical forces—including acceleration, deceleration, rotational forces, or direct impact—acting on the brain (Werner and Engelhard, 2007). This is followed by the secondary injury, driven by cellular and molecular processes that unfold over time (Ng and Lee, 2019). These processes include neuroinflammation, cytokine release, excitotoxicity, oxidative stress, endocrine disruption, and cell death, which may persist for months or even years, exacerbating the initial damage and worsening outcomes (Masel and DeWitt, 2010; Ziebell and Morganti-Kossmann, 2010; Ng and Lee, 2019).

This narrative review focuses specifically on sex hormone dysregulation as one secondary injury-related pathway that may influence two common co-occurring post-TBI complications: post-traumatic epilepsy (PTE) and sleep–wake disturbances. PTE is defined as recurrent, unprovoked seizures occurring more than 1 week after brain injury (Becerra-Hernandez et al., 2025). While seizures can occur immediately or in the days following TBI, these early seizures do not always progress to PTE. The mechanisms that determine whether seizures subside or evolve into chronic epilepsy remain poorly understood. The risk of developing PTE varies by injury severity and location, with incidence rates ranging from approximately 5% to as high as 15–20% (Becerra-Hernandez et al., 2025). About half of the PTE cases begin within the first year post-injury, and 80% occur within the first 2 years.

Sleep disturbances and PTE frequently co-occur after TBI (Orff et al., 2009; Ferguson et al., 2010; Weymann and Rourke, 2021), suggesting that partially overlapping biological processes may contribute to both outcomes. Although the precise mechanisms linking TBI to these outcomes remain under investigation, emerging evidence suggests that sex hormone imbalances after TBI may interact with inflammatory, glial, and excitatory pathways that influence seizure susceptibility and sleep–wake regulation. Rather than providing a systematic review of all endocrine consequences of TBI, we emphasize how hormonal disruption may modulate neuronal and behavioral processes relative to sleep and post-injury seizure risk.

Interpreting human studies in this area is complicated by substantial heterogeneity across TBI cohorts, including differences in injury severity, lesion location, age, sex, baseline sleep habits, and lifestyle factors. Definitions of TBI severity and diagnostic criteria for post-traumatic sleep disorders and epilepsy also vary widely across studies, further limiting the ability to draw consistent mechanistic conclusions. These inconsistencies highlight the need for an integrative framework focused on shared biological pathways—such as hormonal dysregulation—that may contribute to both post-traumatic sleep disturbances and seizure vulnerability.

Endocrine disruptions are common after TBI, yet often remain undiagnosed due to lack of standardized practices for screening or detecting hormonal abnormalities post-injury (Popovic et al., 2005; Richmond and Rogol, 2014; Tanriverdi and Kelestimur, 2015). Secondary injury mechanisms, including hypothalamic and pituitary vulnerability to mechanical forces, can alter multiple hormonal axes (Xiong et al., 2009; Ng and Lee, 2019). A recent comprehensive review of endocrine dysfunction post-TBI calls for improved screening practices and highlights the long-term consequences of untreated hormonal imbalances, particularly involving the hypothalamic–pituitary axis (Mahajan et al., 2023). TBI can also elevate cortisol levels and disrupt hypothalamic-pituitary-gonadal (HPG) axis signaling (Srivastava et al., 2022; Mbiydzenyuy and Qulu, 2024). Elevated cortisol exerts well-established inhibitory effects on the HPG axis by suppressing hypothalamic gonadotropin-releasing hormone (GnRH) release, reducing pituitary responsiveness to luteinizing hormone (LH) and follicle-stimulating hormone (FSH) cues, and directly impairing gonadal steroidogenesis (Tilbrook et al., 2000, Wagenmaker and Moenter, 2017). Chronic or repeated cortisol elevations—common after TBI due to both acute stress responses and hypothalamic vulnerability—can therefore reduce circulating estrogen, progesterone, and testosterone. These interactions suggest that TBI-related HPA axis disruption may secondarily impair sex hormone production, amplifying downstream effects on excitability, glial function, and sleep regulation. Additionally, TBI-induced damage to internal brain structures, including the hypothalamus and pituitary gland, may contribute to hypopituitarism and altered sex hormone production (Zaben et al., 2013; Tanriverdi et al., 2015). Given the heterogeneity of TBI biomechanics and injury locations, endocrine dysfunction varies substantially across individuals.

Sex hormones including estrogen, progesterone, and testosterone are primarily produced in the adrenal glands and gonads but can also be synthesized locally in the brain. Disruption of these hormones is well-documented post-TBI, though the full scope of downstream effects remains unclear (Agha et al., 2004; Tanriverdi and Kelestimur, 2015). Evidence demonstrates that endocrinopathies—defined as long-lasting changes in hormone production, release, circulation, or regulation—occur in more than one-third of TBI patients (Mahajan et al., 2023). Studies, including research from our lab, indicate that experimental TBI can lead to enduring alterations in hormone release, emphasizing the need for a better understanding of these effects and their long-term implications (Rowe et al., 2016; Ortiz et al., 2020; Rowe et al., 2020; Ortiz et al., 2024). Because sex hormones regulate neuroinflammation, synaptic plasticity, glial reactivity, and neuronal excitability, their disruption may contribute to variability in post-TBI sleep patterns and seizure susceptibility. Understanding the mechanistic interplay between these endocrine processes and neural dysfunction is critical for developing new strategies to mitigate chronic post-traumatic complications.

The relationship between sleep and epilepsy is bidirectional: seizures can disrupt sleep architecture, and sleep loss increases cortical excitability. In the TBI context, hormonal dysregulation may further interact with these processes, potentially amplifying vulnerability to sleep fragmentation or seizure expression. Importantly, sex hormones exert well-established influences on sleep–wake behavior, yet females remain underrepresented in sleep research, limiting understanding of sex-specific trajectories (Mannino et al., 2024). Preclinical studies using animal models have provided key insights into how reproductive hormones affect both sleep behavior and seizure susceptibility (Scharfman et al., 2005), although many studies still aggregate data across sexes (Mannino et al., 2024).

Previous reviews have examined sex hormones in TBI recovery (Rubin and Lipton, 2019; Valera et al., 2021), sleep disturbances following brain injury (Mathias and Alvaro, 2012; Viola-Saltzman and Watson, 2012), hormonal influences on epilepsy (Tauboll et al., 2015), and sleep-epilepsy interactions (Nobili et al., 2021). *However, no prior work has synthesized the mechanistic intersections between hormonal dysregulation, sleep dysfunction, and post-traumatic seizure susceptibility*. This integrative framework suggests three possibilities: (1) TBI-induced hormonal changes may represent one interacting factor influencing both sleep and excitability pathways; (2) the established reciprocal relationship between sleep and epilepsy may be exacerbated in the presence of endocrine dysfunction; and (3) sex-specific hormonal trajectories may contribute to variability in sleep and seizure outcomes. By connecting these traditionally separate domains, this review highlights underexplored pathways that may inform earlier identification of hormonal abnormalities and guide future research into therapeutic strategies to improve long-term outcomes after TBI.

## Search strategy and scope

This narrative review was informed by literature identified through searches in PubMed, Google Scholar, and Web of Science between January 2000 and May 2025. Search terms included combinations of “traumatic brain injury,” “sex hormones,” “testosterone,” “estrogen,” “progesterone,” “sleep,” “sleep disturbances,” “epilepsy,” “post-traumatic epilepsy,” and “seizure.” Both preclinical and clinical studies were included, with an emphasis on mechanistic studies relevant to hormone signaling, glial function, and neuroinflammation. Reference lists from relevant articles and prior reviews were also examined to ensure coverage of key findings. Because this is a narrative rather than systematic review, articles were selected for their conceptual relevance to emerging biological themes rather than through predefined inclusion criteria. This review is not intended to be exhaustive, but rather to synthesize evolving evidence and highlight emerging intersections among hormonal dysregulation, sleep disturbances, and post-traumatic seizure susceptibility.

## Hormonal dysregulation after TBI

The effects of sex hormones are mediated through pathways via hormone receptors expressed throughout the brain, including the hypothalamus, hippocampus, and cerebral cortex. Sex hormone dysregulation has been reported following TBI, with implications for inflammation, seizure susceptibility, and sleep disruption. Estrogen, progesterone, and testosterone each modulate glial function and neuronal excitability, and their altered levels post-injury may contribute to both sex-specific outcomes and therapeutic considerations. Understanding how these hormones normally regulate neuroinflammation, synaptic plasticity and glial function provides insight into why their post-injury dysregulation may drive sleep disturbances and seizure susceptibility. Here, we synthesize emerging evidence from both rodent models and human studies on how these hormones are affected by TBI and how they may be leveraged to improve recovery.

Both preclinical and clinical studies indicate that TBI can disrupt hormonal regulation, highlighting the complex interplay between injury-induced changes in the endocrine system and recovery processes. Preclinical studies using rodent models have shown significant alterations in sex hormone levels and receptor expression following TBI. For instance, after mild TBI, male mice exhibit decreased androgen receptor (AR) expression, while female mice show increased estrogen receptor 1 (ESR1) expression. This shift reduces the AR/ESR1 ratio in both sexes, indicating a tilt toward ESR1-driven signaling (Ma et al., 2019). These changes may help explain sex-specific differences in recovery. TBI also disrupts estrous cycling in female rodents, prolonging the diestrus phase and lowering circulating 17β-estradiol levels. In males, repeated TBI causes a sustained drop in testosterone that persists for at least 1 month post-injury (Ma et al., 2019).

Clinical studies corroborate aspects of these findings, revealing variable but often measurable hormone dysregulation following moderate to severe TBI. Recent work shows that post-injury sex hormone profiles may even predict consciousness recovery in patients with severe TBI, suggesting their potential as biomarkers for neural recovery trajectories (Naseri Alavi et al., 2025). In men, TBI typically reduces testosterone levels on the day of injury, while progesterone and estrogen levels rise (Wagner et al., 2011). In contrast, women experience increases in both testosterone and estrogen immediately after injury, with estrogen levels declining more gradually over time. In both sexes, progesterone and testosterone levels usually return to baseline within 6 days (Wagner et al., 2011). Long-term hormonal disturbances are also common; approximately 68% of women with moderate to severe TBI report menstrual irregularities, with hormonal disturbances persisting for several years (Colantonio et al., 2010). Additionally, hypopituitarism, a condition characterized by impaired hormone production, develops in approximately 40% of TBI patients within 3 months post-injury (Mahajan et al., 2023), highlighting the enduring impact of TBI on endocrine function.

Together, these studies demonstrate that TBI can significantly alter the endocrine system, with hormonal imbalances potentially contributing to both acute and long-term injury responses. Identifying and understanding these disruptions is essential for developing targeted interventions that may reduce secondary injury and enhance recovery outcomes.

Estrogen actively reduces neuroinflammation after TBI by enhancing cell survival and promoting neuroprotection (Duncan and Saldanha, 2020). Following brain injury, it supports neurogenesis and recovery by stimulating neural outgrowth and moderating glial activation (Stein, 2001). Inhibiting estrogen synthesis with letrozole significantly elevates glial fibrillary acidic protein (GFAP) levels in female mice post-TBI, indicating increased astrocyte reactivity (Munoz-Ballester and Robel, 2023). Estrogen also lowers pro-inflammatory cytokine levels, limiting leukocyte infiltration, cerebral edema, apoptosis, and astrogliosis (Duncan and Saldanha, 2020). These anti-inflammatory effects are mediated, in part, through estrogen receptor signaling. Activation of estrogen receptor alpha (ERα) improves neurovascular function and facilitates myelin repair after brain injury (Dubal et al., 2001).

Estrogen production is tightly regulated through negative feedback involving the hypothalamus and pituitary gland. TBI-related damage to these regions can impair hormone synthesis and alter circulating estrogen levels, potentially compounding secondary injury and delaying recovery (Khan et al., 2018). While estrogen’s role in modulating glial activation is well established, its influence on long-term outcomes such as seizure susceptibility or cognitive dysfunction requires further investigation, as findings across models and clinical contexts remain heterogeneous.

Like estrogen, progesterone exerts widespread protective effects after TBI, reducing necrotic damage, limiting edema, preventing cell loss, and promoting cognitive recovery (Galani et al., 2001, Shear et al., 2002). Its neuroprotective actions are closely linked to immune modulation, blood–brain barrier stabilization, and support of cellular repair processes. Following injury, progesterone administration reduces expression of pro-inflammatory cytokines such as IL-1β and TNF-α while increasing levels of the anti-inflammatory cytokine IL-10 (Zhou et al., 2024). It also decreases the number of activated microglia and infiltrating neutrophils in the injured cortex and hippocampus, while enhancing splenic regulatory T cell counts and promoting faster edema resolution (Zhou et al., 2024).

At the molecular level, TBI has been suggested to increase expression of the progesterone receptor membrane component 1 (PGRMC1) in neurons and astrocytes, although direct experimental evidence remains limited (Guennoun, 2020). PGRMC1 may mediate progesterone’s protective effects by enhancing glial reactivity, limiting fluid accumulation, and promoting homeostatic recovery. Given limited empirical data, these interpretations should be considered preliminary.

Progesterone also supports post-injury cognitive and functional recovery. In rodent models, it improves spatial memory, reduces lesion volume, and enhances functional outcomes in both males and females (Galani et al., 2001, Guennoun, 2020). These benefits may be partially attributable to progesterone’s ability to modulate excitability through its interactions with GABA_A receptors, which are also involved in sleep regulation and seizure threshold. Although not as well studied as estrogen in this regard, progesterone may exert some of its neuroprotective effects through modulation of GABA_A receptors, potentially contributing to reduced neuronal excitability (Zhou et al., 2024).

Hypogonadism, a common consequence of moderate-to-severe TBI, may result in insufficient progesterone levels in both sexes, worsening secondary injury and delaying recovery (Bavisetty et al., 2008). Despite robust preclinical evidence, two large-scale clinical trials—ProTECT III trial (Wright et al., 2014) and the SyNAPSe trial (Skolnick, Maas et al., 2014)—found no significant improvement in neurological outcomes with intravenous progesterone in patients with moderate-to-severe TBI. These findings suggest that factors such as injury severity, hormone timing and dosage, and sex-specific response profiles may critically influence therapeutic efficacy.

More recent insights propose that the abrupt loss of endogenous progesterone following injury—rather than persistently low levels—may contribute to worse outcomes, particularly in females (Valera et al., 2021). This hypothesis has spurred interest in using hormone trajectory, rather than absolute levels, as a therapeutic guide. Personalized, time-sensitive hormone interventions that align with endogenous hormone dynamics may offer a more effective approach to post-TBI treatment.

Compared to estrogen and progesterone, testosterone has been less extensively studied in the context of TBI. However, emerging preclinical evidence suggests that testosterone plays a critical role in maintaining mitochondrial integrity and neuronal viability, with potential implications for male-specific recovery trajectories. Mitochondrial dysfunction, a hallmark of TBI, leads to elevated intracellular calcium, energy depletion, and apoptosis. Testosterone administration post-TBI restores calcium homeostasis by activating the sodium-calcium-lithium exchanger (NCLX) and stabilizes mitochondrial membrane potential (ΔΨm), which is typically elevated post-injury (Carteri et al., 2019). These findings highlight testosterone’s role in preserving mitochondrial function and limiting secondary cell loss following injury.

Testosterone deficiency is common after moderate-to-severe TBI and can persist for months or longer. This hormonal disruption during critical windows of recovery may impair neuroplasticity, worsen fatigue and depressive symptoms, and contribute to poorer functional outcomes (Carteri et al., 2019). A key mechanistic consideration is that testosterone is locally converted to estradiol via the aromatase enzyme in neurons and glia, and estradiol plays a well-established role in synaptic plasticity, dendritic remodeling, and neuroprotection (Brann et al., 2007). Thus, reduced testosterone availability after TBI may indirectly diminish estradiol-dependent plasticity in injured circuits, particularly in regions where aromatase expression is upregulated as part of the brain’s repair response. Despite its potential, few studies have evaluated testosterone supplementation in clinical TBI populations, and its relationship with chronic neuroinflammation remains largely unexplored. Although testosterone has well-documented immunomodulatory effects in other neurological and peripheral inflammatory conditions (Du et al., 2025), including regulation of microglial reactivity (O'Connor and Nissen, 2023; Du et al., 2025), cytokine production (Mohamad et al., 2019), and mitochondrial stability (Yan et al., 2021), most of this mechanistic evidence comes from non-TBI models. Only a small number of clinical or preclinical studies have directly examined how testosterone influences neuroinflammatory processes specifically after brain injury (Berg et al., 2010; Wagner et al., 2011; Carteri et al., 2019). Thus, while testosterone is a biologically plausible regulator of post-TBI inflammation, its contribution to long-term neuroimmune dysfunction remains an open question requiring targeted investigation.

Estrogen, progesterone, and testosterone each regulate secondary injury mechanisms following TBI, including inflammation, glial activation, and mitochondrial dysfunction. Estrogen and progesterone suppress pro-inflammatory cytokines, reduce astrocyte and microglial activation, and support neurovascular repair (Duncan, 2020, Duncan and Saldanha, 2020, Zhou et al., 2024). Progesterone also promotes GABAergic signaling, which may influence excitability and recovery (Zhou et al., 2024), while testosterone preserves mitochondrial stability and calcium homeostasis (Carteri et al., 2019). These hormones also influence neural excitability, and their post-injury dysregulation may increase seizure susceptibility rather than directly driving epileptogenesis. Importantly, males and females exhibit different hormone trajectories following TBI—such as prolonged testosterone suppression in males and altered estradiol patterns in females—which may shape recovery windows and treatment responsiveness (Rubin and Lipton, 2019; Hoffman et al., 2020). The failure of large-scale hormone-based trials (Wright et al., 2014; Skolnick, Maas et al., 2014) underscores the importance of considering hormone dynamics, not just static levels, when designing future interventions.

## Hormonal effects on post-traumatic epilepsy

The development of hypersynchronous excitability in a previously healthy brain after TBI is complex and not yet fully understood. Hormonal dysregulation is one factor that may interact with inflammatory, metabolic, and circuit-level changes during the post-injury period. Sex hormones such as estrogen and progesterone are known to influence neuronal excitability and seizure susceptibility (Tauboll et al., 2015). For example, in catamenial epilepsy, seizures in women tend to cluster around the perimenstrual and periovulatory phases of the menstrual cycle, when significant fluctuations in estrogen and progesterone levels occur (Herzog, 2008). These fluctuations are thought to drive seizure clustering in catamenial epilepsy, where periovulatory estradiol increases and perimenstrual progesterone withdrawal alter seizure susceptibility (Rogawski, 2003; Herzog et al., 2004; Reddy, 2004; Reddy, 2009; Herzog et al., 2023). During the second half of the follicular phase, estradiol levels peak before ovulation and then rapidly decline, while progesterone rises during the luteal phase and drops before menstruation (Foldvary-Schaefer and Falcone, 2003). These cyclic changes provide a well-established clinical example of how hormonal fluctuations can modulate seizure expression, and they offer a framework for considering how post-TBI endocrine changes might influence excitability.

Progesterone and its metabolites act as anticonvulsants, whereas estrogen and some of its metabolites can enhance excitability depending on dose, receptor subtype, and neural context. Clinical evidence supports these opposing effects: natural progesterone administration reduces seizure frequency in some women with catamenial epilepsy, while a Mendelian randomization study reported that higher estradiol levels were associated with a reduced risk of epilepsy in men (Ke et al., 2023). These findings highlight the complexity and context dependence of hormone–seizure interactions and suggest that post-TBI hormonal shifts may influence seizure susceptibility, although direct evidence in PTE is still limited. To date, no studies have directly demonstrated that TBI-induced hormonal dysregulation causes PTE, and current evidence supports only modulatory roles in seizure susceptibility.

In addition, post-TBI hormone fluctuations may influence biological pathways that underlie both seizure susceptibility and sleep–wake disturbances. Sex hormone dysregulation alters glial activation, neurotransmitter clearance, and inhibitory tone—mechanisms essential not only for controlling neural excitability but also for regulating sleep architecture. Thus, rather than acting through separate mechanisms, hormonal imbalance may serve as a shared upstream vulnerability factor that contributes to both post-traumatic sleep disruption and heightened seizure risk.

Earlier studies also show differential hormonal effects on ictogenesis (seizure triggering). Intravenous conjugated estrogens increased epileptiform activity in a dose-dependent manner in patients with epilepsy (Backstrom, 1976), while intravenous progesterone reduced spike frequency (Mattson et al., 1984). In rodent models, systemic testosterone administration lowered the seizure threshold, suggesting a potential proconvulsant effect (Edwards et al., 1999). Collectively, these studies demonstrate that sex hormones can modulate seizure expression in already epileptic brains, though their relevance to epileptogenesis—the development of new epilepsy after TBI—remains incompletely defined. Given that TBI frequently induces hormonal dysregulation, it is plausible that endocrine changes may interact with other injury-induced processes to modulate PTE risk. Clarifying this relationship may help identify early intervention windows.

Beyond direct effects on neuronal excitability, sex hormones also modulate glial function, particularly astrocytic regulation of extracellular glutamate and GABA, two neurotransmitters critical for maintaining excitatory/inhibitory (E/I) balance. In a rat model of ischemia, estrogen and progesterone significantly increased the expression of astrocytic glutamate transporters GLT-1 and EAAT3, reduced infarct volume, and improved behavioral outcomes, suggesting that enhanced glutamate clearance may underlie their neuroprotective effects (Nematipour et al., 2020). Although ischemia and TBI are distinct injuries, both involve excitotoxicity and glial activation; thus, these mechanisms may hold relevance for TBI but require direct validation. Additional work in seizure and injury models shows that estradiol and progesterone upregulate EAAT expression and modulate metabotropic glutamate receptor activity, thereby influencing excitatory transmission (Goyette et al., 2023).

Separate lines of evidence suggest that neurosteroids modulate GABAergic signaling, potentially affecting transporters like GAT-3, which maintains inhibitory tone (Mincheva et al., 2022). While hormonal control of GAT-3 has not been demonstrated specifically in TBI, cortical injury downregulates GAT-3 in thalamic astrocytes, leading to network hyperexcitability and increased seizure susceptibility. Enhancing GAT-3 function in this context reduces hyperexcitability (Cho et al., 2022). These findings suggest mechanistic pathways through which hormone-related disruptions could interact with glial dysfunction after TBI, but the specific contribution of hormonal dysregulation to these changes remains an open question.

Clinical studies further support a systemic connection between sex hormones and neurotransmitter regulation: circulating glutamate levels fluctuate with estrogen and progesterone levels across the menstrual cycle (Zlotnik et al., 2011). Preclinical models of ischemic brain injury show that estrogen increases GLT-1 and EAAT3 expression in both astrocytes and neurons, enhancing glutamate reuptake and mitigating excitotoxic damage (Gazzaley et al., 1996; Liang et al., 2002; Hernández-Jiménez et al., 2016). Together, these findings support the hypothesis that hormone dysregulation after TBI may contribute to impaired glutamate and GABA buffering—one of several processes that could create a pro-epileptogenic environment—though direct evidence linking hormone disruption to PTE development is still emerging.

## Hormonal contributions to post-TBI sleep disturbances

Sleep disturbances are a common long-term consequence of TBI. Over 50% of individuals report experiencing some form of sleep disruption after a TBI, with 25–29% diagnosed with a specific sleep disorder—rates significantly higher than those of the general population (Mathias and Alvaro, 2012). These disorders include hypersomnia, sleep apnea, narcolepsy, periodic limb movement disorder, and parasomnia (Viola-Saltzman and Watson, 2012; Rowe and Griesbach, 2022). Additionally, individuals post-TBI are two to four times more likely to experience issues such as sleepwalking, early awakenings, excessive sleepiness, nightmares, and reduced sleep maintenance and efficacy (Mathias and Alvaro, 2012). Although sleep disturbances arise from multiple injury-related processes—including neuroinflammation, circuit dysregulation, and structural damage—we highlight hormonal dysregulation as one factor that may contribute to this vulnerability.

In women, sleep quality is influenced by hormonal fluctuations during the menstrual cycle (Baker and Driver, 2007; Baker and Lee, 2018). Women generally report better sleep during the follicular and ovulatory phases compared to the premenstrual phase, which is characterized by a dramatic decrease in estrogen and progesterone (Brown et al., 2008). Sleep disturbances, including insomnia and poor sleep quality, worsen in the late luteal and premenstrual phases (Baker and Lee, 2018). For example, one study reported increased slow-wave sleep during the luteal phase, when progesterone concentrations peak (Baker et al., 2001), while another study found poorer sleep quality during the late luteal phase, when progesterone levels drop (Jia et al., 2024). A population-based survey also found that women using hormonal contraceptives had better sleep efficiency and a lower apnea–hypopnea index compared to naturally cycling women (Hachul et al., 2013). These observations illustrate how shifts in estrogen and progesterone can influence sleep architecture and sleep quality under normal physiological conditions.

Animal studies further support the role of sex hormones on sleep regulation. In female mice, administration of 17β-estradiol reduced non-rapid eye movement (NREM) sleep, suggesting that estrogen may modulate sleep architecture (Paul et al., 2009). In contrast, male mice that underwent gonadectomy and received testosterone treatment showed increased NREM sleep. In humans, testosterone levels in men naturally rise with sleep onset, peak during the first rapid eye movement (REM) episode, and remain elevated until waking (Luboshitzky et al., 1999). Delayed REM onset is associated with slower increases in testosterone, while low testosterone levels are linked to sleep deprivation, fragmentation, and shorter sleep duration (Hirokawa et al., 2022). Sleep fragmentation can also disrupt testosterone rhythms—men with fragmented sleep exhibit impaired testosterone regulation (Luboshitzky et al., 2001). Notably, men who sleep fewer than 6 h have significantly lower testosterone levels than those who sleep more than 8 h (Goh and Tong, 2010). These findings support a bidirectional relationship between sex hormones and sleep regulation.

**Figure 1:**
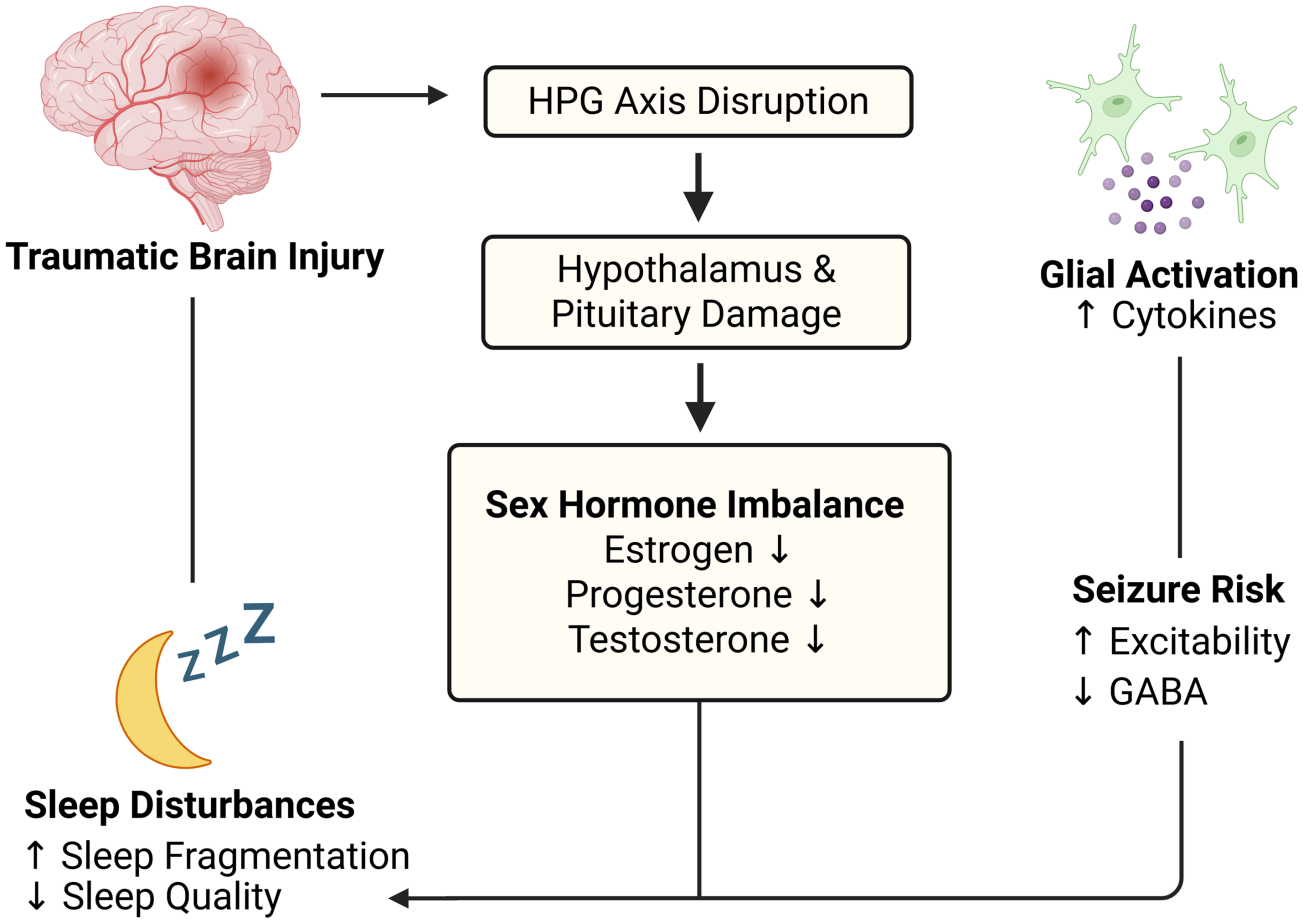
Sex hormone dysregulation as a potential convergent mechanism linking TBI to sleep and seizure outcomes. Traumatic brain injury (TBI) disrupts the hypothalamic–pituitary–gonadal (HPG) axis via mechanical injury and stress-related endocrine signaling, altering circulating and brain-derived estrogen, progesterone, and testosterone. These hormonal changes may modulate glial activation, neurotransmission, and neuroinflammatory pathways implicated in both sleep–wake disruption and seizure susceptibility. Although causality has not been established, converging evidence suggests hormone dysregulation may create a shared physiological environment that increases vulnerability to both outcomes. Bidirectional interactions between sleep disturbance and seizure activity may further amplify post-injury neural instability. This figure summarizes hypothesized pathways and highlights potential therapeutic targets, underscoring the need for future studies to define mechanistic links among hormone signaling, sleep regulation, and epileptogenesis after TBI.

The clear relationship between sex hormones and sleep, demonstrated in numerous studies, highlights the potential mechanistic link between hormonal dysregulation and sleep disturbances (Howell and Griesbach, 2024). In a recent preclinical study, sex differences in sleep architecture were observed following TBI, with female rodents exhibiting reductions in REM sleep, resulting in impaired episodic memory—findings that suggest hormone-dependent differences in vulnerability but do not yet establish direct causal pathways (Howell and Griesbach, 2024). Translating these results to clinical contexts, it is plausible that hormone disruptions following TBI may interact with other secondary injury mechanisms to contribute to sleep dysfunction. Investigating this link further could inform early, hormone-targeted interventions aimed at preventing sleep disturbances and improving recovery outcomes. Together, these findings support the hypothesis that post-TBI hormonal dysregulation may be one component of a shared pathophysiological environment that influences both sleep and seizure disturbances (Figure 1).

## The reciprocal relationship between epilepsy and sleep

PTE and sleep disturbances are both common secondary consequences of TBI and share a reciprocal relationship: poor sleep can increase seizure susceptibility, while epileptic activity and seizures can degrade sleep quality. People with epilepsy are significantly more likely to experience sleep disorders than those without epilepsy, with insomnia and obstructive sleep apnea being the most frequently reported (Peng et al., 2021). These associations suggest that sleep and seizure activity influence one another in ways that may also be relevant after TBI, although most existing data come from non-TBI epilepsy populations. Disrupted sleep impairs the brain’s ability to regulate seizures, while seizures themselves—and the antiepileptic medications used to treat them—can further disturb sleep architecture (Nobili et al., 2021). Nighttime seizures frequently interrupt sleep cycles, and daytime seizures often impair sleep quality the following night. Several seizure triggers are also tightly linked to sleep, including sleep deprivation, seizures that occur exclusively during sleep, and seizures provoked by transitions between sleep and wakefulness (Ng and Pavlova, 2013). These phenomena reflect ictogenesis (the triggering of seizures), which is distinct from epileptogenesis, the long-term process of developing epilepsy.

Electroencephalography (EEG) studies illustrate the importance of sleep in modulating seizure expression. NREM sleep facilitates seizure activity, whereas REM sleep tends to exert inhibitory effects on cortical synchrony (Nobili et al., 2022). A systematic review found that epileptic spikes occurring during REM sleep could help localize epileptic zones in 84% of the cases analyzed (Nobili et al., 2022). Seizure-induced changes in sleep parameters, such as decreased REM sleep quantity and delays in the onset of the first REM episode, have also been documented. Additionally, decreased sleep spindle density, particularly surrounding epileptic foci, has been observed in patients with drug-resistant epilepsy (Nobili et al., 2022) suggesting that seizures can disrupt features of sleep linked to memory and cortical stability.

Recent research has begun to explore molecular interactions between sleep and epilepsy. Sleep is regulated by circadian rhythms controlled by the suprachiasmatic nucleus and peripheral clocks. Altered expression of circadian clock proteins has been identified in some epileptic tissues, including reduced Clock gene expression in focal epilepsy samples (Jin et al., 2020). In animal models, deletion of the Clock gene in mice lowered seizure thresholds and increased spontaneous epileptiform discharges in excitatory neurons (Li et al., 2017). Additional studies show decreased Clock, Per1, and Per2 mRNA expression in the anterior hypothalamus of epileptic mice (Wallace et al., 2018). Together, these findings suggest that circadian and sleep-regulatory pathways may interact with neural excitability, though the specific mechanisms linking these processes—particularly in the context of TBI—remain to be clarified.

## Conclusion and research gaps

Brain injuries are known to induce changes in sex hormone concentrations, with the degree of disruption influenced by the severity and location of the injury. These hormonal changes, particularly in estrogen, testosterone, and progesterone, have systemic effects on the central nervous system, yet their downstream molecular and circuit-level consequences remain incompletely defined. Sex hormone dysregulation is also a recognized feature in disorders such as epilepsy and sleep disturbances. While evidence suggests a bidirectional relationship between hormonal fluctuations and the expression or exacerbation of these disorders, direct causal links between TBI-induced hormonal changes and the development of PTE or post-traumatic sleep disturbances have not been firmly established. In this review, we brought together findings from endocrine physiology, sleep research, and epilepsy neuroscience to evaluate whether sex hormone dysfunction may represent a convergent mechanism influencing both post-traumatic seizure vulnerability and sleep–wake disruption—an integrative question that has not been addressed in prior reviews.

Despite extensive work demonstrating that sex hormones can modulate seizure susceptibility and sleep regulation under normal physiological or disease conditions, the specific mechanisms by which post-TBI hormonal changes interact with secondary injury processes—such as neuroinflammation, glial activation, mitochondrial dysfunction, and network instability—remain poorly understood. Recent reviews have emphasized the need to identify molecular biomarkers and risk factors for PTE, including inflammatory signaling, glial remodeling, and, potentially, endocrine disruption (Kazis et al., 2024). These observations suggest that sex hormones may function as upstream modulators of excitability rather than isolated causal drivers of epileptogenesis. Our synthesis highlights how these endocrine-driven pathways overlap with those implicated in sleep fragmentation, offering a conceptual framework in which hormone dysregulation after TBI may contribute to both outcomes through shared biological processes.

Several key research gaps remain: (1) The relative contribution of TBI-induced hormone dysregulation to ictogenesis versus epileptogenesis is unknown; (2) The interactions between hormone trajectories (rather than single timepoint measurements) and evolving sleep architecture after TBI have not been systematically studied; (3) Few studies have examined whether hormone therapies timed to endogenous fluctuations could mitigate post-TBI sleep disturbances or seizure risk; (4) Mechanistic work linking endocrine changes to glial buffering of glutamate and GABA after TBI is limited; (5) Sex-specific pathways remain severely understudied, despite clear differences in hormone dynamics and sleep outcomes. Identifying these mechanistic intersections was a central goal of this review, and the gaps outlined here provide a roadmap for the studies required to advance the field.

Addressing these gaps will be essential for determining whether sex hormones contribute to a shared biological environment that influences both sleep disturbances and seizure susceptibility after TBI. Future studies incorporating longitudinal endocrine assessments, sex-stratified analyses, mechanistic modeling, and targeted intervention trials will be critical for clarifying whether hormone-informed therapeutic strategies can improve long-term neurological outcomes. By articulating how endocrine disruption may intersect with sleep and excitability pathways, this review fulfills its objective of defining sex hormone dysfunction as a potentially unifying factor in chronic post-traumatic neurological complications.

### Limitations and future directions

While substantial evidence demonstrates that sex hormones modulate excitability, sleep regulation, and inflammatory responses, the mechanisms by which TBI-induced hormonal changes influence these processes remain only partially understood. Several limitations in the current literature constrain interpretation. Human studies vary widely in definitions of TBI severity, sleep disorders, and epilepsy, and outcomes are influenced by confounding factors such as age, comorbidities, medication use, sleep habits, and lifestyle. Variability in injury biomechanics and lesion location further complicates efforts to link endocrine disruption with specific neural or behavioral outcomes. At the molecular level, hormones exert context-dependent effects shaped by receptor availability and local enzymatic activity, making it challenging to generalize findings across models or individuals.

Preclinical models, while essential for mechanistic insight, face their own limitations. Isolating the effects of individual hormones is difficult due to the interconnected nature of endocrine signaling, and few studies have systematically manipulated hormone receptors or metabolic enzymes in a TBI context. Likewise, although sex hormones can influence astrocytic glutamate and GABA transporters, direct evidence linking hormonal dysregulation to impaired glial buffering after TBI is scarce. Recent work implicating GAT-3 and GLT-1 in post-injury hyperexcitability highlights the need to determine whether hormone fluctuations modulate these transporters or associated glial pathways.

Future progress will require coordinated clinical and preclinical approaches. Human studies would benefit from standardized criteria for TBI severity, sleep disturbances, and epileptic events, along with longitudinal designs that track hormone trajectories rather than single timepoints. Simultaneous EEG and endocrine monitoring represents a particularly promising opportunity, as no studies have yet mapped how evolving hormone profiles interact with sleep architecture or ictal activity during the latent period after injury. In parallel, animal models incorporating receptor- or enzyme-specific manipulations could clarify how sex hormones intersect with neuroinflammatory, metabolic, and glial pathways implicated in epileptogenesis and sleep dysfunction.

Finally, although early hormone-based interventions after TBI have yielded mixed clinical results, hormone-informed therapeutic strategies remain worth exploring. Rather than relying on static hormone replacement, future approaches may need to align with endogenous hormone trajectories, sex differences, and critical windows after injury. Neurosteroid-based compounds such as ganaxolone, allopregnanolone analogs, and progesterone receptor modulators have shown promise in improving excitability and sleep regulation in other neurological conditions and may offer a path forward for TBI (Reddy and Estes, 2016; Sperling et al., 2017; Meltzer-Brody et al., 2018; Knight, Amin et al., 2022). In parallel, sex-specific hormone replacement strategies and selective estrogen receptor modulators are being investigated as tools to stabilize neuroinflammation, glial signaling, and neurotransmitter homeostasis (Vegeto et al., 2003; Chakrabarti et al., 2014; Lu et al., 2025). Agents that enhance glial transporter function or modulate estrogen and progesterone signaling in a targeted manner represent additional promising avenues but require rigorous testing across both sexes and injury severities. Together, these emerging therapeutic directions highlight that clarifying the role of hormone dysregulation after TBI is not only mechanistically important but may directly inform future interventions for post-traumatic sleep and seizure disorders.

Together, addressing these limitations and exploring these future directions will be essential for determining whether sex hormones contribute to a shared biological landscape influencing both sleep disturbances and seizure susceptibility after TBI.
